# Multi-omics analyses of the gut microbiota and metabolites in children with metabolic dysfunction-associated steatotic liver disease

**DOI:** 10.1128/msystems.01148-24

**Published:** 2025-03-14

**Authors:** Landuoduo Du, Kaichuang Zhang, Lili Liang, Yi Yang, Deyun Lu, Yongchang Zhou, Tianyi Ren, Jiangao Fan, Huiwen Zhang, Ying Wang, Lu Jiang

**Affiliations:** 1 Division of Pediatric Gastroenterology and Nutrition, Xinhua Hospital, Shanghai Jiao Tong University School of Medicine, Shanghai, China; 2 Department of Clinical Nutrition, College of Health Science and Technology, Shanghai Jiao Tong University School of Medicine, Shanghai, China; 3 Department of Pediatric Endocrinology and Genetic Metabolism, Xinhua Hospital, Shanghai Jiao Tong University School of Medicine, Shanghai, China; 4 Shanghai Institute for Pediatric Research 674079 , Shanghai, China; 5 Shanghai Key Laboratory of Pediatric Gastroenterology and Nutrition 633081 , Shanghai, China; 6 Department of Gastroenterology, Xinhua Hospital, Shanghai Jiao Tong University School of Medicine, Shanghai, China; APC Microbiome Ireland, Cork, Ireland

**Keywords:** steatosis, pediatric, *Ruminococcus torques*, deoxycholic acid, biomarker

## Abstract

The development and severity of metabolic dysfunction-associated steatotic liver disease (MASLD) in children are closely related to alterations of gut microbiota. This study aims to investigate changes in the gut microbiota signature and microbial metabolites in children with MASLD. We collected fecal samples from children and adolescents aged 6–16 years, and the presence of MASLD was diagnosed by ultrasound. We performed 16S ribosomal DNA sequencing and targeted metabolomics in 36 and 25 subjects, consisting of healthy controls, children with obesity, and children with MASLD. The α-diversity was significantly lower in children with obesity and MASLD compared with healthy controls. Linear discriminant analysis of effect size analysis identified *Anaerostipes* and *A. hadrus* as the top biomarkers differentiating the obesity group from the MASLD group. In MASLD patients with high alanine aminotransferase values (≥50 U/L for boys and 44 U/L for girls), we observed a decrease in the gut microbiota health index. MASLD patients with high shear wave elastography (*E*) values (≥6.2 kPa) showed an increased abundance of *Ruminococcus torques*, which was positively correlated with the levels of deoxycholic acid (DCA) and *E* values. Importantly, the mediation analysis identified positive associations between *R. torques* and clinical indicators of MASLD that were mediated by DCA. Overall, our study suggests that gut microbiota and metabolites are significantly altered in children with MASLD, and targeting *R. torques* may offer potential benefits for disease management.

**IMPORTANCE:**

This study investigated alterations in the gut microbiota signature and microbial metabolites in children with metabolic dysfunction-associated steatotic liver disease (MASLD). We found that an increased abundance of *Ruminococcus torques* was associated with increased levels of deoxycholic acid and the progression of MASLD, suggesting that *R. torques* may serve as a novel clinical target in pediatric MASLD.

## INTRODUCTION

Metabolic dysfunction-associated steatotic liver disease (MASLD) has emerged as the most common cause of chronic liver disease worldwide, with a global prevalence of ~25% (1). MASLD is defined by the presence of liver steatosis with at least one cardiometabolic risk factor, which is not required in the non-alcoholic fatty liver disease (NAFLD) definition (2). The clinical spectrum of MASLD ranges from simple steatosis to metabolic dysfunction-associated steatohepatitis with varying amounts of liver fibrosis, which may progress to cirrhosis (3). In children and adolescents, the occurrence of MASLD is often linked to metabolic disorders such as obesity and insulin resistance (3). Currently, there is no effective treatment available, and the mechanisms underlying its pathophysiology remain unknown.

Gut microbiota have been implicated in several aspects during the pathogenesis of MASLD through the “gut-liver” axis (4). Previous studies showed a reduced gut microbial diversity in children with MASLD when compared with healthy controls (5, 6). Specifically, the abundance of *Oscillospira*, *Blautia*, and *Faecalibacterium* was reduced in the fecal samples of children with MASLD, while *Prevotella*, *Anaerococcus*, and *Dorea* were increased (6–8). By metabolomics, Luo et al. (5) identified two novel gut metabolites, creatinine and dodecanoic acid, that were elevated in children with NAFLD. Furthermore, their levels were significantly correlated with pathways associated with lipid metabolism, inflammation, and impaired liver function. By integrating metagenomics with metabolomics, Chierico et al. (7) showed that a decrease in *Oscillospira* accompanied by an increased level of 2-butanone could serve as gut microbiota signatures for children with steatosis. Therefore, it is of great clinical value to identify key gut microbiota and microbial metabolites that may influence the progression of pediatric MASLD.

In this study, we performed 16S ribosomal DNA (rDNA) sequencing and targeted metabolomics in a well-characterized pediatric MASLD cohort. By exploring associations between features of gut microbiota and severity of pediatric MASLD, our study identified novel biomarkers that could potentially facilitate the non-invasive diagnosis of disease and may serve as clinical targets.

## MATERIALS AND METHODS

### Human subjects

Children and adolescents (*n* = 36 for 16S rDNA sequencing and *n* = 25 for targeted metabolomics) ranging in age from 6 to 16 years were recruited for this study. Obese children were recruited from the Department of Pediatric Endocrinology and Genetic Metabolism of Xinhua Hospital, meeting the criteria of a *z*-score of body mass index (BMI) > +2 SD according to the World Health Organization (9). Obese patients were further divided into two groups: obesity or MASLD. Inclusion criteria for children with MASLD were the presence of liver steatosis by ultrasound diagnosis, the absence of steatogenic drugs, the absence of diseases causing secondary steatosis, and the absence of other chronic liver diseases as previously stated (10).

Healthy controls were recruited from volunteers with *z*-scores of BMI < +1 SD. The use of antibiotics or probiotics within the 3 months prior to sampling was excluded. Demographic, clinical, and laboratory parameters were obtained from available subjects.

### Bacterial DNA extraction and 16S rDNA gene sequencing

Bacterial DNA was extracted from human stool samples using a TIANamp stool DNA kit (TIANGEN, Beijing, China). The extracted DNA was subjected to electrophoresis on a 1% (wt/vol) agarose gel. The concentration and purity of DNA were determined using a NanoDrop 2000 UV-visible spectrophotometer (Thermo Scientific, Wilmington, USA). The full-length bacterial 16S rDNA gene was amplified using the universal bacterial primers 27F (5′-AGRGTTYGATYMTGGCTCAG-3′) and 1492R (5′-RGYTACCTTGTTACGACTT-3′) (11–13). The PCR products were purified using AMPure PB beads (Pacific Biosciences, CA, USA) and quantified with Qubit 4.0 fluorometer (Thermo Fisher Scientific, USA). The purified PCR products were pooled in equimolar concentrations, and a DNA library was constructed using the SMRTbell prep kit 3.0 (Pacific Biosciences, CA, USA). The purified library was then sequenced on the PacBio Sequel IIe System (Pacific Biosciences) by Majorbio Bio-Pharm Technology Co., Ltd. (Shanghai, China).

Raw reads from PacBio were demultiplexed, and HiFi reads were generated using the circular consensus sequencing mode of the SMRT Link analysis software (version 11.0) with a minimum of three full passes and 99% sequence accuracy. The HiFi reads were then subjected to barcode identification and length filtering. Sequences shorter than 1,000 bp or longer than 1,800 bp were excluded. HiFi reads were clustered into operational taxonomic units (OTUs) with a 97% sequence similarity using the UPARSE 11 software (14, 15). For each OTU, the most abundant sequence was selected as its representative sequence. Taxonomic classification of OTUs was performed using the ribosomal database project(RDP) classifier against the Silva 16S rRNA gene database with a 70% confidence threshold.

### Targeted metabolomics profiling and preprocessing

Targeted metabolomics was conducted using ultra-performance liquid chromatography coupled to tandem mass spectrometry (UPLC-MS/MS) system (ACQUITY UPLC-Xevo TQ-S, Waters Corp., Milford, MA, USA). The Q300 Kit (Metabo-Profile Corp. Shanghai, China) was utilized for fecal metabolomics profiling, encompassing a comprehensive range of up to 310 metabolites and 12 biochemical classes, as previously described (16).

Briefly, approximately 5 mg of each lyophilized fecal sample was weighed and transferred to a 1.5 mL tube. After adding 25 µL of water, the sample was homogenized with zirconium oxide beads for 3 minutes. Following homogenization, 120 µL of methanol containing internal standards was added to extract metabolites, followed by another 3 minutes of homogenization and centrifugation at 18,000 × *g* for 20 minutes. Next, 20 µL of the supernatant was transferred to each well in a 96-well plate, followed by the addition of 20 µL of freshly prepared derivatization reagent, with the plate then sealed and incubated at 30°C for 60 minutes. Following derivatization, the samples were diluted with 50% cold methanol solution, stored at −20°C for 20 minutes, and centrifuged at 4,000 × *g* for 30 minutes at 4°C. Finally, 135 µL of the supernatant was transferred to a new 96-well plate, with 10 µL of internal standards added to each well. The resulting supernatant, mixed with internal standards for each sample, was sealed prior to UPLC-MS/MS analysis. Quality control samples were generated by pooling all the samples to be analyzed on the instrument.

The raw data generated by UPLS-MS/MS were processed using TMBQ software (v1.0, Human Metabolomics Institute, Shenzhen, Guangdong, China) for the quantification, calibration, and peak integration of each metabolite (17). Statistical analyses were performed using the self-developed platform iMAP (v1.0, Metabo-Profile, Shanghai, China).

### Real-time qPCR

Bacterial genomic DNA was extracted from human fecal samples as described above. The primer sequences for *Ruminococcus torques* were 5′-GCTTAGATTCTTCGGATGAAGAGGA-3′ (forward) and 5′-AGTTTTTACCCCCGCACCA-3′ (reverse) (18). The primer sequences for 16S were 5′-GTGSTGCAYGGYTGTCGTCA-3′ (forward) and 5′- ACGTCRTCCMCACCTTCCTC-3′ (reverse), as previously described (19). Real-time qPCR was performed using SYBR Premix (Applied Biosystems) as previously reported (20). The detection of *R. torques* was performed using the following cycling conditions: an initial denaturation at 95°C for 5 minutes, followed by 40 cycles of 95°C for 10 seconds and 60°C for 30 seconds. The relative expression of *R. torques* was normalized to 16S.

### Statistical analysis

Clinical characteristics are presented as median and range unless specified otherwise. Comparisons between the two groups were conducted using the *t*-test or the Mann-Whitney *U* test for nonparametric data. Comparisons among three or more groups were performed using one-way analysis of variance with Tukey’s post hoc test for parametric data or the Kruskal-Wallis test for nonparametric data followed by Dunn’s post hoc test. Multiple comparisons were adjusted using the false discovery rate (FDR) method. Categorical variables were assessed using Fisher’s exact test. All statistical tests were two-sided, with a significance level set at *P* < 0.05.

The similarities of microbial community structure between samples were examined by principal coordinates analysis (PCoA) based on the Bray-Curtis distance algorithm using the “vegan” package in R (v2.4.3) (21). Permutational multivariate analysis of variance (PERMANOVA) test was performed with 999 permutations. The gut microbiome health index (GMHI) was used to predict the likelihood of disease as previously described (22). For each sample, the “collective abundance” of health-prevalent species ($\psi M_{H}$) and health-scarce species ($\psi M_{N}$) was determined. For the species in sets $M_{H}$ and $M_{N}$ in the same sample i, the GMHI can be calculated as follows:


$$h_{i,M_{H},M_{N}}=\log10\left(\frac{\psi M_{H},i}{\psi M_{N},i}\right).$$

In this formula, “$h_{i,M_{H},M_{N}}$” denotes the degree to which sample i portrays the collective abundance of $M_{H}$ to that of $M_{N}$. To reflect the degree of microbial dysbiosis, the microbial dysbiosis index (MDI) was calculated as the log of “total abundance in genera increased in disease group” over “total abundance in genera decreased in disease group” (23). To identify the specific bacterial taxa associated with MASLD (ranging from phylum to species levels), we performed linear discriminant analysis (LDA) effect size (LEfSe) analysis (24) (http://huttenhower.sph.harvard.edu/LEfSe) with the cutoff of LDA > 2 and *P* < 0.05. The mediation analysis was performed in subjects with both gut microbiota and metabolomics data to infer the potential role of the microbial features contributing to MASLD through metabolites via the “mediate” function from the R package “mediation” (version 4.5.0). The differential metabolites were used as candidate mediators. The Benjamini–Hochberg method for multiple testing correction was employed for the average causal mediation effect(ACME) *q*, with a threshold of FDR < 0.2.

In targeted metabolomics, principal component analysis (PCA) was applied to cluster metabolites from the control, obesity, and MASLD groups. Variable importance in projection (VIP) values was obtained based on the orthogonal projection to latent structures-discriminant analysis (OPLS-DA) methods. Metabolites with VIP of ≥1 and *P* < 0.05 were regarded as statistically significant. The *z*-score represents the number of SDs by which an observation deviates above or below the mean value of the control group.

### STORMS checklist

This study has been completed according to the STORMS checklist (DOI: https://doi.org/10.5281/zenodo.14545287).

## RESULTS

### Study population

16S rDNA sequencing and targeted metabolomics were conducted on fecal samples. A cohort of 36 subjects was recruited to explore the gut microbiota signature, including 14 healthy controls, 12 children with obesity, and 10 children with MASLD. The median age of children with MASLD was 11.50 years (range 8.00–16.00 years), with 90% being male, and a median BMI was 28.15 kg/m^2^ (range 22.58–39.60 kg/m^2^). The demographics and clinical data are summarized in Table 1. In comparison to obese patients, children with MASLD exhibited significantly higher rates of arterial hypertension and elevated levels of aspartate aminotransferase (AST), alanine aminotransferase (ALT), gamma-glutamyltransferase (GGT), albumin, fasting glucose, and uric acid (Table 1).

**TABLE 1:** Clinical characteristics of the cohort for 16S rDNA sequencing^*a*^

Parameter	Control	Obesity	MASLD	*P*
Total (*n*)	14	12	10	
Demographics				
Age, years	10.00 (6.00–13.00)	8.50 (6.00–13.00)	11.50 (8.00–16.00)	0.025
Gender, male, *n* (%)	7 (50.00)	8 (66.67)	9 (90.00)	0.133
BMI, kg/m^2^	17.57 (14.79–18.11)	24.30 (19.90–41.50)	28.15 (22.58–39.60)	<0.001
Type 2 diabetes, *n* (%)		0 (0.00)	3 (30.00)	0.078
Arterial hypertension, *n* (%)		0 (0.00)	4 (40.00)	0.029
Metabolic syndrome, *n* (%)		3 (25.00)	5 (50.00)	0.378
Metformin use, *n* (%)		0 (0.00)	1 (10.00)	0.455
Laboratory parameters				
AST, U/L		24.00 (17.00–29.00)	36.00 (22.00–108.00)	0.007
ALT, U/L		21.00 (13.00–27.00)	77.50 (22.00–190.00)	0.004
GGT, U/L		15.00 (9.00–27.00)	43.50 (21.00–110.00)	0.006
Alkaline phosphatase, U/L		303.00 (221.00–381.00)	257.50 (96.00–407.00)	0.155
Total bilirubin, μmol/L		8.20 (5.00–14.80)	11.75 (4.7–76.2)	0.360
Direct bilirubin, μmol/L		2.50 (1.40–3.70)	3.30 (1.20–9.80)	0.096
Albumin, g/L		45.50 (42.60–51.10)	47.70 (46.60–55.00)	0.018
Triglycerides, mmol/L		1.12 (0.54–2.10)	1.27 (0.91–4.77)	0.205
Total cholesterol, mmol/L		4.17 (3.35–5.46)	4.19 (3.09–5.79)	0.895
HDL cholesterol, mmol/L		1.35 (0.95–1.86)	1.14 (0.93–1.37)	0.034
LDL cholesterol, mmol/L		2.59 (1.80–3.60)	2.57 (1.60–3.66)	0.664
Fasting glucose, mmol/L		4.46 (3.62–5.58)	5.02 (4.07–11.12)	0.020
HbA1c, %		5.50 (5.00–5.80)	5.60 (5.10–12.40)	0.303
Alpha-fetoprotein, ng/mL		1.24 (0.00–6.33)	1.19 (0.00–2.28)	0.643
Creatinine, μmol/L		34.00 (29.00–49.00)	38.50 (25.00–71.00)	0.480
Urea, mmol/L		5.34 (2.54–6.59)	4.61 (3.03–6.38)	0.615
Uric acid, μmol/L		348.00 (264.00–533.00)	506.50 (300.00–813.00)	0.010
White blood cell count, ×10^9^/L		7.16 (4.46–10.40)	6.49 (5.26–10.39)	0.692
C-reactive protein, mg/L		0.00 (0.00–8.00)	0.00 (0.00–35.00)	0.853
Platelet count, ×10^9^/L		339.00 (259.00–469.00)	334.50 (222.00–423.00)	0.553

^*a*^ Values presented are median with a range in parentheses for continuous variables or number and percentage in parentheses for categorical variables. AST, aspartate aminotransferase; ALT, alanine aminotransferase; GGT, gamma-glutamyltransferase; HDL cholesterol, high-density lipoprotein cholesterol; LDL cholesterol, low-density lipoprotein cholesterol.

In the cohort for targeted metabolomics, the median age of children with MASLD was 13.00 years (range 8.00–16.00 years), all of whom were male, with a median BMI of 29.74 kg/m^2^ (range 26.40–42.95 kg/m^2^). Compared with children with obesity, children with MASLD had significantly higher rates of arterial hypertension and elevated levels of AST, ALT, GGT, and high-density lipoprotein (HDL) cholesterol, as shown in Table 2.

**TABLE 2:** Clinical characteristics of the cohort for targeted metabolomics^*a*^

Parameter	Control	Obesity	MASLD	*P*
Total (*n*)	7	7	11	
Demographics				
Age, years	8.00 (6.00–13.00)	9.00 (6.00–13.00)	13.00 (8.00–16.00)	0.020
Gender, male, *n* (%)	6 (85.71)	6 (85.71)	11 (100.00)	0.303
BMI, kg/m^2^	16.57 (15.80–18.11)	26.80 (19.90–41.50)	29.74 (26.40–42.95)	<0.001
Type 2 diabetes, *n* (%)		0 (0.00)	3 (27.27)	0.245
Arterial hypertension, *n* (%)		0 (0.00)	7 (63.64)	0.013
Metabolic syndrome, *n* (%)		2 (28.57)	8 (72.73)	0.145
Metformin use, *n* (%)		0 (0.00)	1 (9.09)	1.000
Laboratory parameters				
AST, U/L		23.50 (17.00–29.00)	31.00 (19.00–287.00)	0.024
ALT, U/L		21.00 (16.00–27.00)	77.00 (17.00–347.00)	0.009
GGT, U/L		16.00 (9.00–27.00)	48.00 (21.00–123.00)	0.002
Alkaline phosphatase, U/L		295.00 (221.00–356.00)	243.00 (89.00–407.00)	0.330
Total bilirubin, μmol/L		8.05 (5.30–13.70)	8.50 (4.70–76.20)	0.801
Direct bilirubin, μmol/L		2.55 (1.60–3.60)	2.60 (1.20–9.80)	0.481
Albumin, g/L		45.65 (42.60–51.10)	47.10 (41.40–50.60)	0.595
Triglycerides, mmol/L		1.44 (0.57–2.10)	1.41 (0.62–6.30)	0.920
Total cholesterol, mmol/L		4.31 (3.35–5.46)	4.46 (3.02–5.65)	0.929
HDL cholesterol, mmol/L		1.33 (0.95–1.86)	1.07 (0.76–1.37)	0.040
LDL cholesterol, mmol/L		2.70 (1.80–3.54)	3.01 (1.60–3.66)	0.631
Fasting glucose, mmol/L		4.54 (4.15–5.58)	5.03 (3.88–15.50)	0.191
HbA1c, %		5.55 (5.40–5.80)	5.50 (5.10–12.40)	0.761
Alpha-fetoprotein, ng/mL		1.24 (0.00–1.72)	1.26 (0.00–3.52)	0.816
Creatinine, μmol/L		36.00 (29.00–49.00)	41.00 (29.00–71.00)	0.200
Urea, mmol/L		5.37 (2.54–6.59)	4.47 (2.85–6.21)	0.261
Uric acid, μmol/L		333.50 (299.00–533.00)	460.00 (300.00–813.00)	0.119
White blood cell count, ×10^9^/L		7.25 (4.46–10.40)	7.39 (5.15–14.70)	0.964
C-reactive protein, mg/L		3.00 (0.00–8.00)	1.00 (0.00–35.00)	0.611
Platelet count, ×10^9^/L		346.00 (259.00–469.00)	324.00 (222.00–423.00)	0.192

^*a*^ Values presented are median with a range in parentheses for continuous variables or number and percentage in parentheses for categorical variables. AST, aspartate aminotransferase; ALT, alanine aminotransferase; GGT, gamma-glutamyltransferase; HDL cholesterol, high-density lipoprotein cholesterol; LDL cholesterol, low-density lipoprotein cholesterol.

### Decreased microbial diversity and altered gut microbiota composition in children with obesity and MASLD

**Fig 1:**
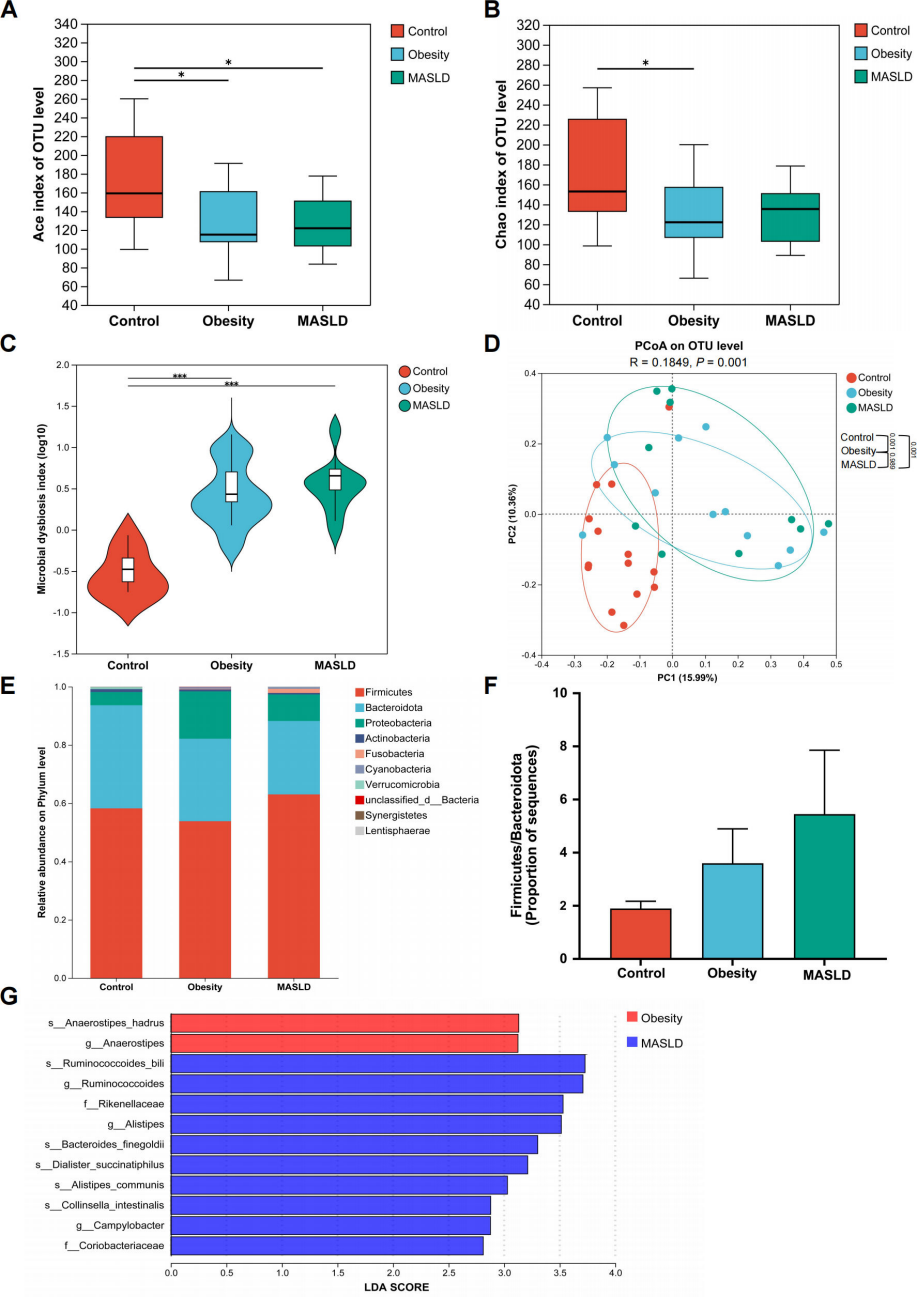
Decreased microbial diversity and altered gut microbiota composition in children with obesity and MASLD. (**A and B**) Alpha diversity was estimated based on the Ace (**A**) and Chao index (**B**) among the healthy controls (*n* = 14), children with obesity (*n* = 12), and children with MASLD (*n* = 10). (**C**) The MDI was calculated for the three groups. (**D**) PCoA was performed at the OTU level. The PERMANOVA *P* value was calculated with 999 permutations. (**E**) Relative abundance of bacteria at the phylum level among the three groups. (**F**) The Firmicutes/Bacteroidota ratio among the three groups. (**G**) LEfSe analysis of the gut microbiota in patients from the obesity and MASLD groups. *P < 0.05, **P < 0.01, and ***P < 0.001.

By 16S rDNA sequencing, we generated an average of 41,772 reads per sample (minimum = 28,544 reads, maximum = 55,706 reads, and median = 42,542 reads). The α-diversity was significantly lower in the obesity group compared with the control group, while no significant difference was observed between the obesity group and the MASLD group, as indicated by the Ace index (Fig. 1A) and Chao index (Fig. 1B). Consistently, the MDI was significantly higher in the obesity and MASLD groups compared with the controls, reflecting a greater degree of bacterial disturbance (Fig. 1C). The PCoA plot showed distinct clustering patterns among the control, obesity, and MASLD groups. The PERMANOVA test revealed significant differences between the control and obesity groups (*P* = 0.001) and between the control and MASLD groups (*P* = 0.001), while no significant difference was observed between the obesity and MASLD groups (Fig. 1D).

At the phylum level, the relative abundance of Fusobacteria was significantly higher in both the obesity and MASLD groups compared with the control group (*P* = 0.044). Although no statistically significant differences were observed, an increasing trend in the Firmicutes/Bacteroidota ratio was evident in both the obesity and MASLD groups (Fig. 1F). LEfSe analysis was performed to identify biomarkers in the obesity and MASLD groups. *Anaerostipes hadrus* and *Anaerostipes* were identified as significant biomarkers in the obesity group, while *Ruminococcoides* was identified as the top biomarker in the MASLD group (Fig. 1G).

To investigate age-related differences in gut microbiota, we further stratified the MASLD cohort into two subgroups: children (8–11 years; *n* = 5) and early adolescents (12–16 years; *n* = 5). Although no significant differences in α-diversity were observed between the two groups (Fig. S1A and B), early adolescents exhibited lower GMHI values and higher MDI values (Fig. S1C and D). Similarly, β-diversity showed no significant differences between the groups (Fig. S1E). The microbial composition at the phylum level showed a higher proportion of Bacteroidota in the children group, although the difference was not statistically significant (Fig. S1F). Using LEfSe analysis, we identified potential biomarkers for MASLD children, including *Faecalibacterium prausnitzii* and the genus *Faecalibacterium* (Fig. S1G).

### Association of gut microbiota composition with ALT

ALT is a commonly used predictive marker for the presence of MASLD in the absence of other causes of liver injury (3). Therefore, we next stratified MASLD patients into two groups based on their ALT values: high ALT (ALT ≥ 50 U/L for boys and 44 U/L for girls) and low ALT (ALT < 50 U/L for boys and 44 U/L for girls) (25). When comparing the gut microbiota composition between patients with obesity and MASLD stratified by ALT values, no significant differences in α-diversity were observed, as indicated by the Ace index (Fig. S2A) and Sobs index (Fig. S2B). The MDI was higher in the MASLD group compared with the obesity group, regardless of ALT values (Fig. S2C). Furthermore, the PCoA plot revealed no differences among the three groups (Fig. S2D). The GMHI was lower in the high ALT group compared with the low ALT group (Fig. S2E). At the phylum level, no significant differences were observed among the three groups (Fig. S2F). LEfSe analysis showed that *Collinsella intestinalis* was the only biomarker in the MASLD group with high ALT (Fig. S2G).

### Association of gut microbiota composition with liver stiffness

**Fig 2:**
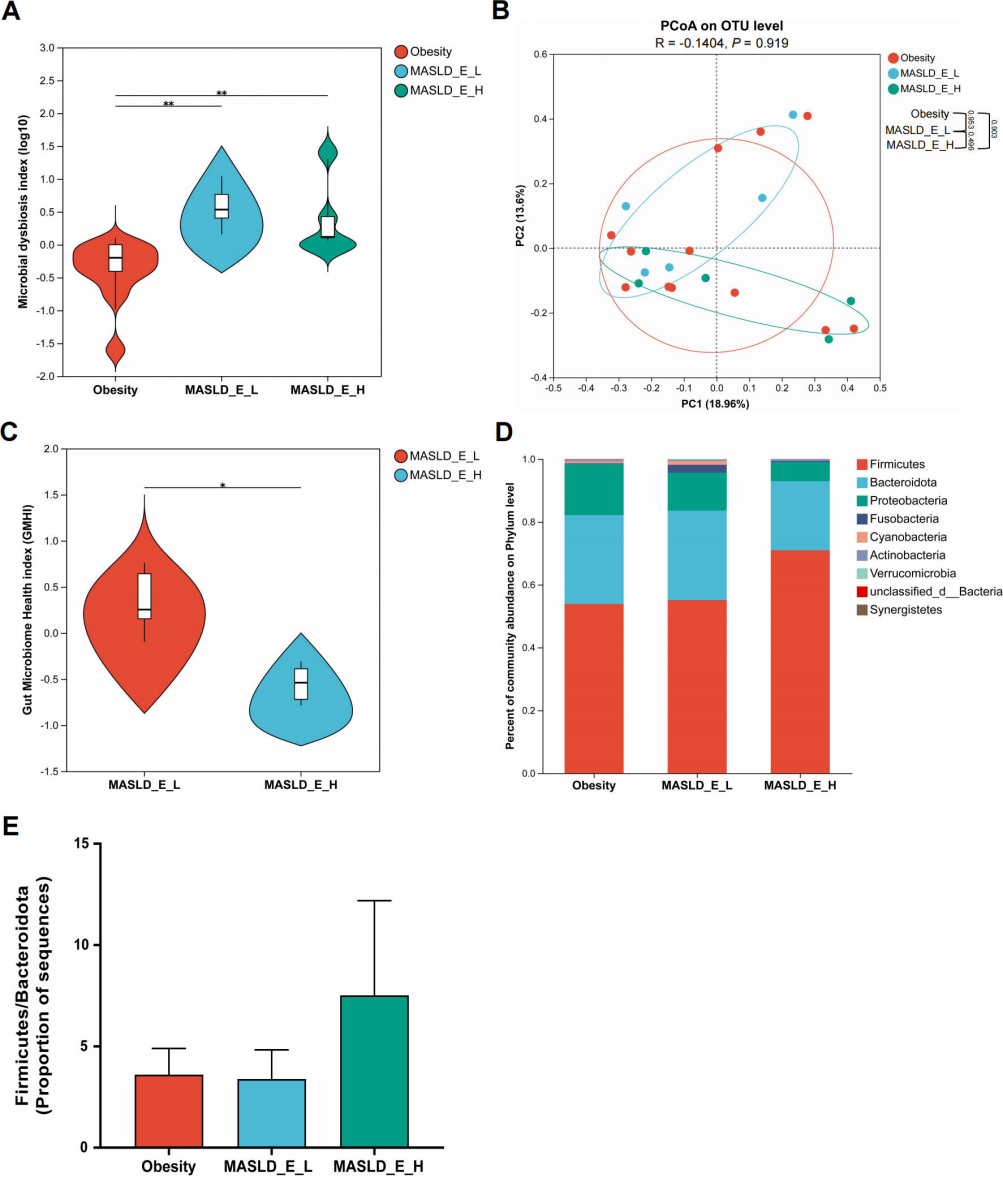
Association of gut microbiota composition with shear wave elastography values. (**A**) The MDI was calculated to reflect the degree of bacterial disturbance among the obesity group (*n* = 12), the low *E* group (*n* = 5, *E* < 6.2 kPa), and the high *E* group (*n* = 5, *E* ≥ 6.2 kPa). (**B**) The PCoA plot was used to show beta diversity among the three groups at the OTU level. The PERMANOVA *P* value was calculated with 999 permutations. (**C**) The GMHI was calculated between the low *E* group and the high *E* group to assess the health status of the gut microbiota. (**D**) Relative abundance of bacteria at the phylum level among the three groups. (**E**) The Firmicutes/Bacteroidota ratio among the three groups. *P < 0.05 and **P < 0.01.

**Fig 3:**
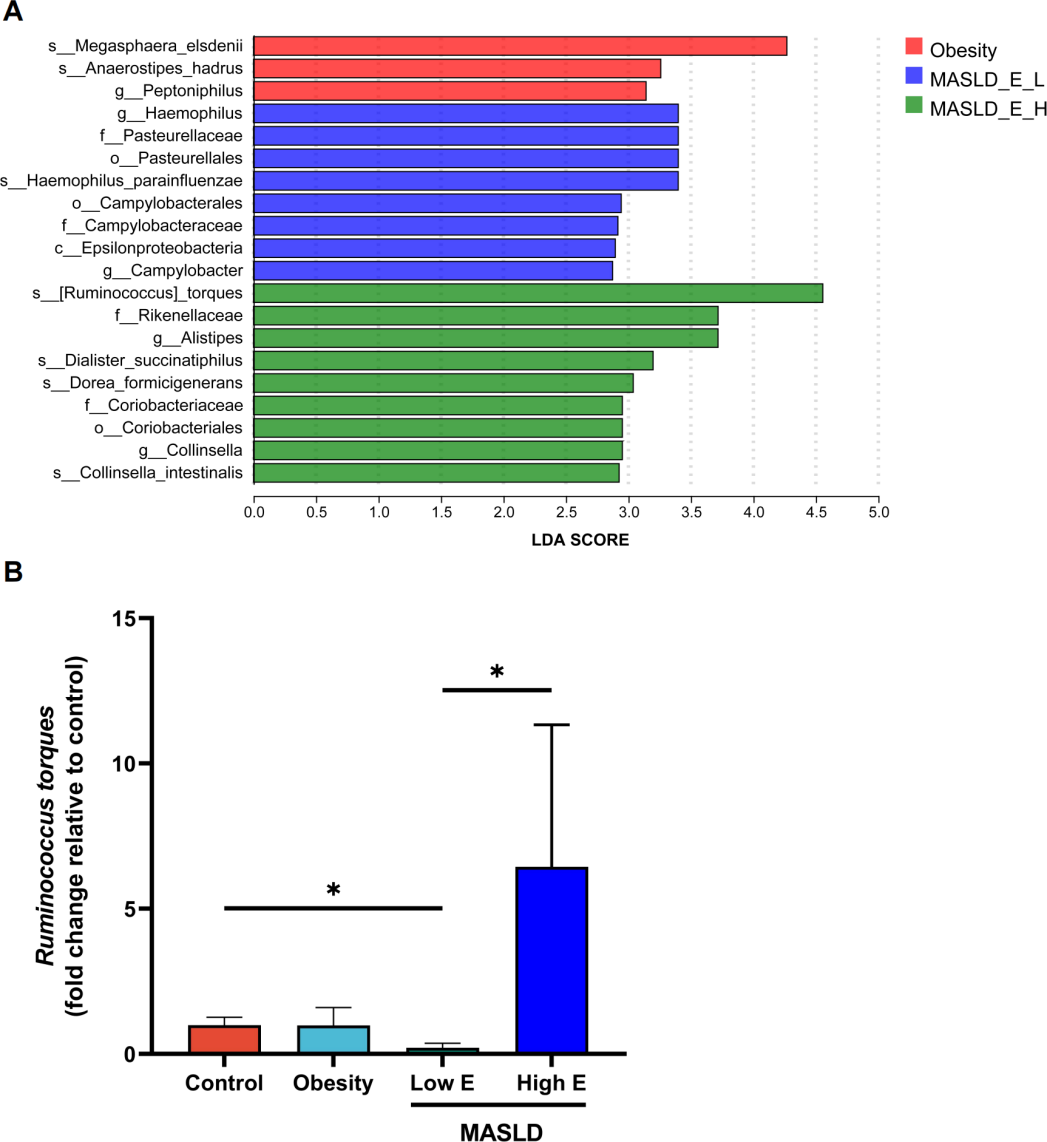
Increased abundance of *R. torques* in MASLD patients with high shear wave elastography values. (**A**) LEfSe analysis of gut microbiota among the obesity group (*n* = 12), the low *E* group (*n* = 5), and the high *E* group (*n* = 5). (**B**) Fecal levels of *R. torques* assessed by qPCR. *P < 0.05.

In children with MASLD, liver stiffness was assessed using shear wave elastography, and patients were stratified into two groups based on the upper limit of the reference range for normal hepatic elastography (*E*) values (2.6–6.2 kPa) (26). When comparing obese children with MASLD patients stratified based on *E* values (high *E*: ≥6.2 kPa vs low *E*: <6.2 kPa), the MASLD group had a higher MDI compared to the obesity group, with no difference between the high *E* and low *E* groups (Fig. 2A). PCoA analysis revealed no distinction among the three groups (Fig. 2B). Importantly, the GMHI was lower in the high E group, indicating a higher likelihood of disease presence (Fig. 2C). At the phylum level, no significant differences were observed among the three groups (Fig. 2D). Although no statistically significant differences were detected, an increasing trend in the Firmicutes/Bacteroidota ratio was observed in the MASLD group, particularly in the high *E* subgroup (Fig. 2E). By LEfSe analysis, *R. torques* was identified as the top biomarker in MASLD patients with high *E* values (Fig. 3A). Last, we examined the levels of *R. torques* in fecal samples from the control, obesity, and MASLD groups by qPCR. As expected, the abundance of *R. torques* was significantly higher in MASLD patients with high *E* values compared to those with low *E* values. No significant differences in abundance were observed between the control and obesity groups or between the obesity and low *E* groups (Fig. 3B). Collectively, these findings suggest a potential association between the abundance of *R. torques* and the increased level of liver stiffness in pediatric MASLD.

### Altered fecal metabolomic profile in children with MASLD

**Fig 4:**
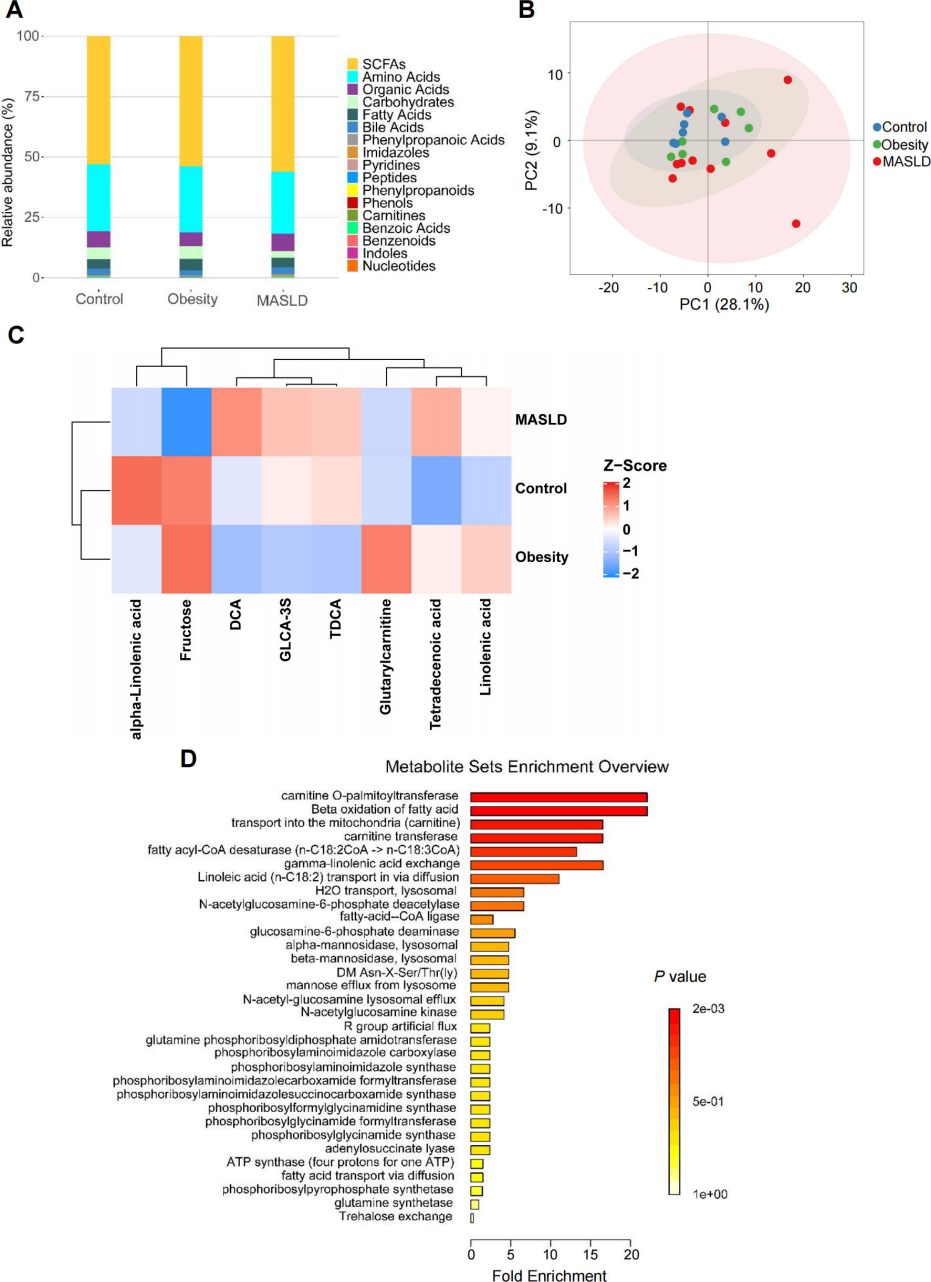
Altered fecal metabolomic profile in children with MASLD. (**A**) Relative abundance (%) of the metabolites among the healthy controls (*n* = 7), the obesity group (*n* = 7), and the MASLD group (*n* = 11). (**B**) The PCA plot was used to show the similarities and differences among the three groups. (**C**) The heatmap analysis showing the eight differential metabolites among the three groups. (**D**) Metabolite set enrichment analysis of differential metabolites.

To investigate the gut microbial metabolites associated with pediatric MASLD progression, targeted metabolomics was conducted on fecal samples from 25 subjects, comprising 7 healthy controls, 7 children with obesity, and 11 children with MASLD. A total of 213 metabolites across 17 classes were identified, including well-known gut microbiota-derived metabolites, such as short-chain fatty acids, amino acids, organic acids, carbohydrates, fatty acids, and bile acids (Fig. 4A; Table S1). The PCA plot displayed a similar clustering pattern among the three groups (Fig. 4B). The heatmap showed the eight differential metabolites among the three groups (Fig. 4C). Metabolite set enrichment analysis of differentiating metabolites revealed enrichment of multiple pathways associated with lipid metabolism, such as carnitine o-palmitoyltransferase, beta-oxidation of fatty acid, transport into the mitochondria (carnitine), and carnitine transferase (Fig. 4D).

**Fig 5:**
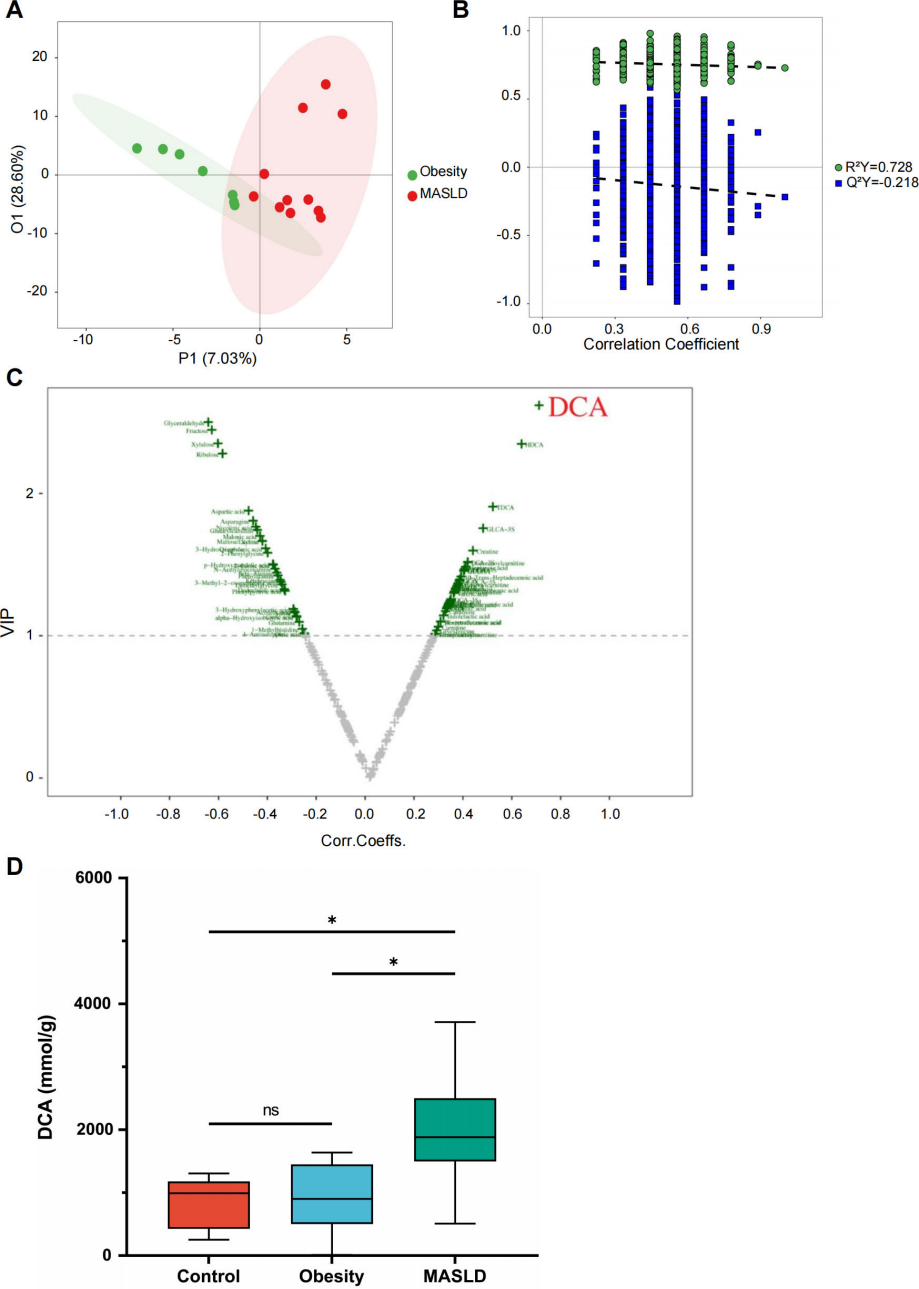
Increased levels of DCA in children with MASLD. (**A and B**) OPLS-DA plot (**A**) and permutation test (**B**) between the obesity (*n* = 7) and the MASLD groups (*n* = 11). (**C**) Volcano plot showing the VIP values based on the OPLS-DA model. (**D**) The level of DCA in healthy controls (*n* = 6), the obesity group (*n* = 7), and the MASLD group (*n* = 10). *P < 0.05.

To identify differential metabolites between the obesity and MASLD groups, OPLS-DA was applied to obtain VIP values (Fig. 5A through C). Notably, we found that deoxycholic acid (DCA) was the metabolite with the highest VIP score (Fig. 5C). When comparing the MASLD group with the obesity group, we observed that the levels of DCA were significantly higher in the MASLD group (Fig. 5D). These observations suggest that the increased levels of DCA could be a key metabolite in the pathogenesis of pediatric MASLD.

### Correlations between the gut microbiota and metabolites in MASLD

**Fig 6:**
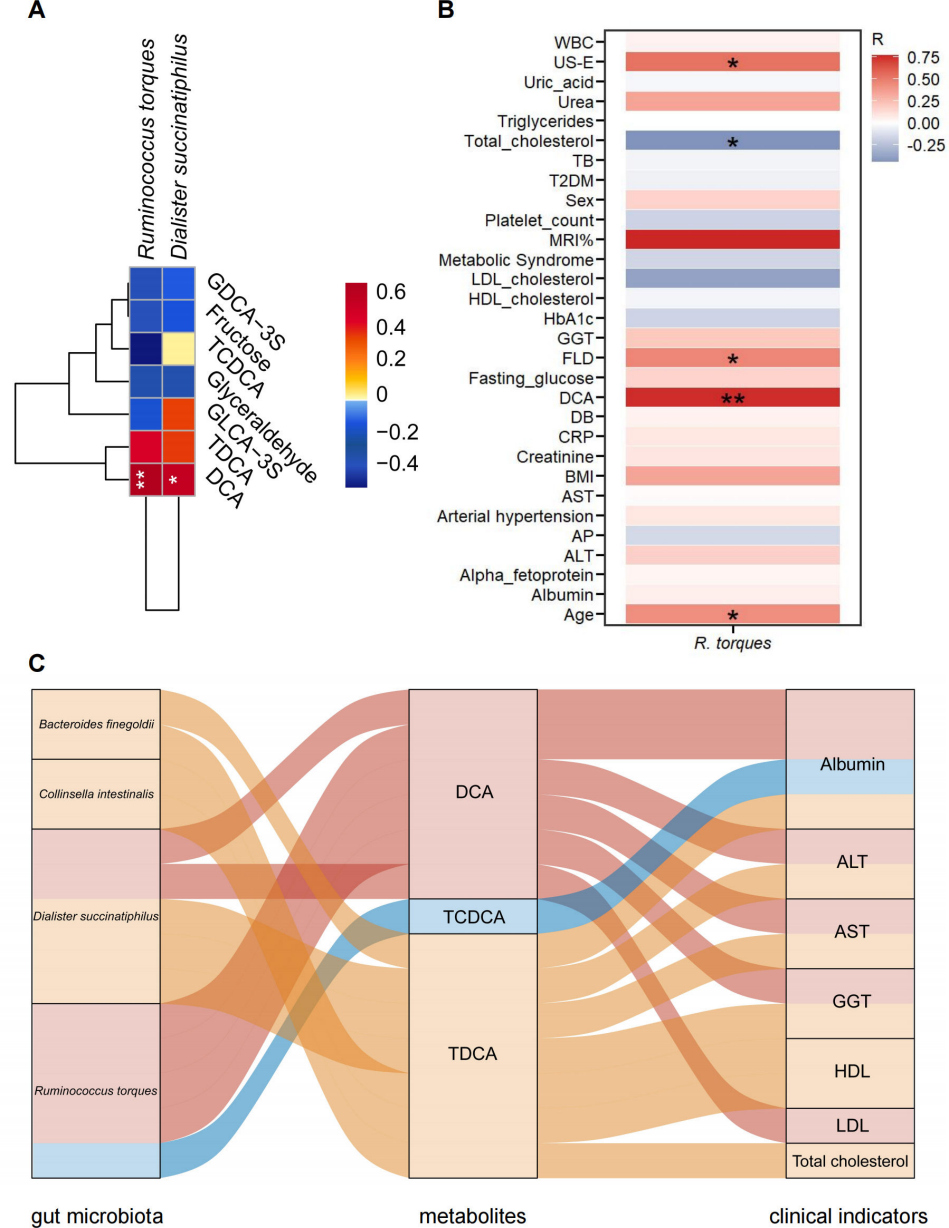
Correlations between the gut microbiota and metabolites in children with obesity and MASLD. (**A**) Heatmap of Spearman’s correlation between the seven differential metabolites and significantly altered bacteria at the species level when comparing the MASLD group (*n* = 11) with the obesity group (*n* = 7). (**B**) Heatmap of Spearman’s correlation between the abundance of *R. torques* and clinical parameters. (**C**) Sankey plots showing the correlation network between the gut microbiota and the clinical indicators mediated by fecal metabolites (*q* value < 0.2). Curved lines connecting panels represent mediation effects, with colors corresponding to different mediators. GDCA-3S, glycodeoxycholic acid 3-sulfate; GLCA-3S, glycolithocholic acid 3-sulfate. *P < 0.05 and **P < 0.01.

By correlation analysis, we found that the levels of DCA were positively correlated with the abundance of *R. torques* (*R* = 0.751) and *Dialister succinatiphilus* (*R* = 0.640), as shown in Fig. 6A. Furthermore, heatmap analysis revealed a positive correlation among the abundance of *R. torques*, DCA levels, the presence of FLD, and US-*E* values (Fig. 6B). Finally, we performed mediation analyses using the differential microbiota between the obesity and MASLD groups, with metabolites serving as potential mediators (Fig. 6C). A total of 14 linkages were identified mediating the associations between gut microbiota and clinical indicators of MASLD through metabolites, including DCA, taurochenodeoxycholic acid, and taurodeoxycholic acid. Consistently, *R. torques* was positively associated with clinical indicators of MASLD, mediated through DCA (Fig. 6C). Taken together, these data suggest that increased levels of *R. torques* might be an important contributor to DCA elevation, eventually leading to the progression of pediatric MASLD.

## DISCUSSION

Alterations of gut microbiota and metabolites are closely related to the progression of MASLD in both children and adults (27). In this study, we examined the gut microbiota profile and fecal metabolites in a well-characterized cohort of obese children with or without MASLD. Overall, we found a reduced microbial diversity in children with obesity and MASLD. Further analysis identified *A. hadrus* as the top biomarker distinguishing obesity from the MASLD groups. As the top biomarker in MASLD patients with high E values, the abundance of *R. torques* was positively correlated with both DCA levels and *E* values. By mediation analysis, we highlighted the significant mediating roles of metabolites in the association between gut microbiota and pediatric MASLD.

In our study, we compared the gut microbiota signature among the control, obesity, and MASLD groups. Interestingly, we found no significant difference in α-diversity between children with obesity and those with MASLD. Consistently, Schwimmer et al. (6) compared the gut microbiota between children with obesity (controls) and those with NAFLD at different stages. Interestingly, they found that while α-diversity was lowest in children with non-alcoholic steatohepatitis (controls, 3.52; NAFLD, 3.36; borderline NASH, 3.37; NASH, 2.97; *P* = 0.001), there was no significant difference between the control group and the NAFLD or borderline NAFLD groups. As liver biopsies were not performed in our study, we could not stratify the MASLD group into various stages accurately, which may have influenced the α-diversity results. Furthermore, since obesity is a major risk factor for MASLD, the microbiome changes associated with obesity might overlap substantially with those seen in the MASLD group, potentially explaining the lack of difference in α-diversity between the two groups. Therefore, a larger cohort of patients with confirmed biopsy results is necessary to confirm our observations regarding microbial diversity in pediatric MASLD.

Previous studies have consistently shown an increased abundance of Proteobacteria in adult patients with NAFLD/MASLD (28–30). In contrast, we observed an expansion of Proteobacteria in children with obesity compared with the control group, while its abundance did not further increase in the MASLD group. Moreover, its abundance was comparable among the obesity group and MASLD groups with both low and high *E* values. Interestingly, by comparing the gut microbiota between children with metabolic dysfunction-associated fatty liver disease (MAFLD) and healthy controls, Ji et al. (31) showed that the proportion of Proteobacteria was similar between the two groups (9.85% in controls vs 10.26% in MAFLD). As pediatric studies on gut microbiota to date are still limited and may be influenced by factors such as ethnicity, geography, and diet, drawing firm conclusions remains challenging.

By comparing the differential bacteria between the obesity and MASLD groups, we identified *Anaerostipes* and *A. hadrus* as the biomarkers for the obesity group. *Anaerostipes*, a genus within the Firmicutes phylum, is known for containing species that produce butyrate (32). As the dominant species within this genus, *A. hadrus* is considered beneficial in the human gut due to its butyrate production (33). However, recent studies suggest that *A. hadrus* exhibited detrimental effects in mice with colitis induced by dextran sulfate sodium while showing no impact on healthy mice (33). Furthermore, a recent multi-omics study revealed that fatty acid biosynthesis could be mediated by *A. hadrus*, impacting the availability of long-chain free fatty acids in the portal circulation and thereby promoting hepatic fibrosis. In accordance with our findings (data not shown), *A. hadrus* was found to be depleted in adults with progressive NAFLD (NASH, fibrosis, and cirrhosis) (34). Therefore, further work is required to establish the causal relationship between *A. hadrus* and the progression of pediatric MASLD.

One interesting finding is that *R. torques* was the top biomarker in MASLD patients with high *E* values, and its fecal levels were significantly higher compared with those with low *E* values. Furthermore, a positive correlation was observed between fecal levels of *R. torques* and DCA, indicating that DCA could be a product of *R. torques*. This aligns with the study from Wu et al. (35), which demonstrated that an increased abundance of *R. torques* in mice led to elevated DCA levels. Given the positive correlation between *R. torques* and *E* values, it is reasonable to hypothesize that *R. torques* may promote the progression of pediatric MASLD by producing DCA. Surprisingly, gavage with *R. torques* improved high-fat diet (HFD)-induced obesity, glucose homeostasis, and insulin sensitivity in mice by increasing DCA levels, resulting in G-protein-coupled bile acid receptor (GPBAR1/TGR5) activation and elevation of white adipose tissue thermogenesis. These results parallel earlier discoveries documented by Gillard et al. (36), who demonstrated that administering DCA to *foz/foz* mice on a HFD restored bile acid levels in portal blood, increased TGR5 and farnesoid X receptor (FXR) signaling, ameliorated dysmetabolic conditions, prevented steatosis and hepatocellular ballooning, and decreased macrophage infiltration (36). Considering that the etiological factors contributing to MASLD progression are different between children and adults, it is possible that *R. torques* exhibits differential effects. In a study that characterized the gut microbiota composition in patients with Crohn’s disease (CD) and their relatives, they showed that the abundance of *R. torques* was significantly increased in relatives of patients with CD (37). Given that *R. torques* is known to degrade gastrointestinal mucin, the higher colonization of this bacterium could potentially precede or predispose individuals to the development of CD (18). Collectively, current research indicates that *R. torques* may exert its effects through various mechanisms, such as producing metabolites like DCA or degrading gastrointestinal mucin. Future research is needed to fully understand the causative role of *R. torques* and its underlying mechanisms in pediatric MASLD.

The strength of our study is the integration of 16S rDNA sequencing with targeted metabolomics in a well-characterized pediatric MASLD cohort, thus providing a comprehensive understanding of gut microbiota and its metabolites in the progression of disease. A shortcoming of our study is the small sample size. Future studies are warranted to validate our results in a larger group of patients. Additionally, our study is limited by the lack of complete controlled attenuation parameter (CAP) data for all patients. Future studies with complete CAP data will be better positioned to explore the potential associations between gut microbiota and liver steatosis. As our study did not include NAFLD cases that fall outside the MASLD criteria, future research is needed to explore and compare the differences in gut microbiota signatures between NAFLD and MASLD.

In conclusion, our study demonstrated a decrease in microbial diversity in children with obesity and MASLD. In children with MASLD, an increased abundance of *R. torques* is associated with elevated liver stiffness and DCA levels. By comprehensively analyzing the gut microbiota and microbial metabolites in pediatric MASLD, our study offers valuable insights into potential therapeutic strategies for disease management.

## Data Availability

The data sets generated and analyzed in the course of this research are accessible upon formal request to the corresponding author. Raw sequencing data from 16S rDNA sequencing were registered at NCBI under BioProject PRJNA1130618.

## SUPPLEMENTAL MATERIAL

Fig. S1 and S2 msystems.01148-24-s0001.docx Alterations in the microbial diversity and gut microbiota composition associated with age and ALT level in MASLD groups.

Table S1 msystems.01148-24-s0002.xlsx Metabolomics data.

ASM does not own the copyrights to Supplemental Material that may be linked to, or accessed through, an article. The authors have granted ASM a non-exclusive, world-wide license to publish the Supplemental Material files. Please contact the corresponding author directly for reuse.
