# Supplementary material for: Multi-omics analyses of the gut microbiota and metabolites in children with metabolic dysfunction-associated steatotic liver disease
Source: mSystems. 2025 Mar 14;10(4):e01148-24. doi: 10.1128/msystems.01148-24 (PMC12013275; doi:10.1128/msystems.01148-24)
Supplement: Fig. S1 and S2 — Alterations in the microbial diversity and gut microbiota composition associated with age and ALT level in MASLD groups. [file msystems.01148-24-s0001.docx]

**Supplemental Figure 1**


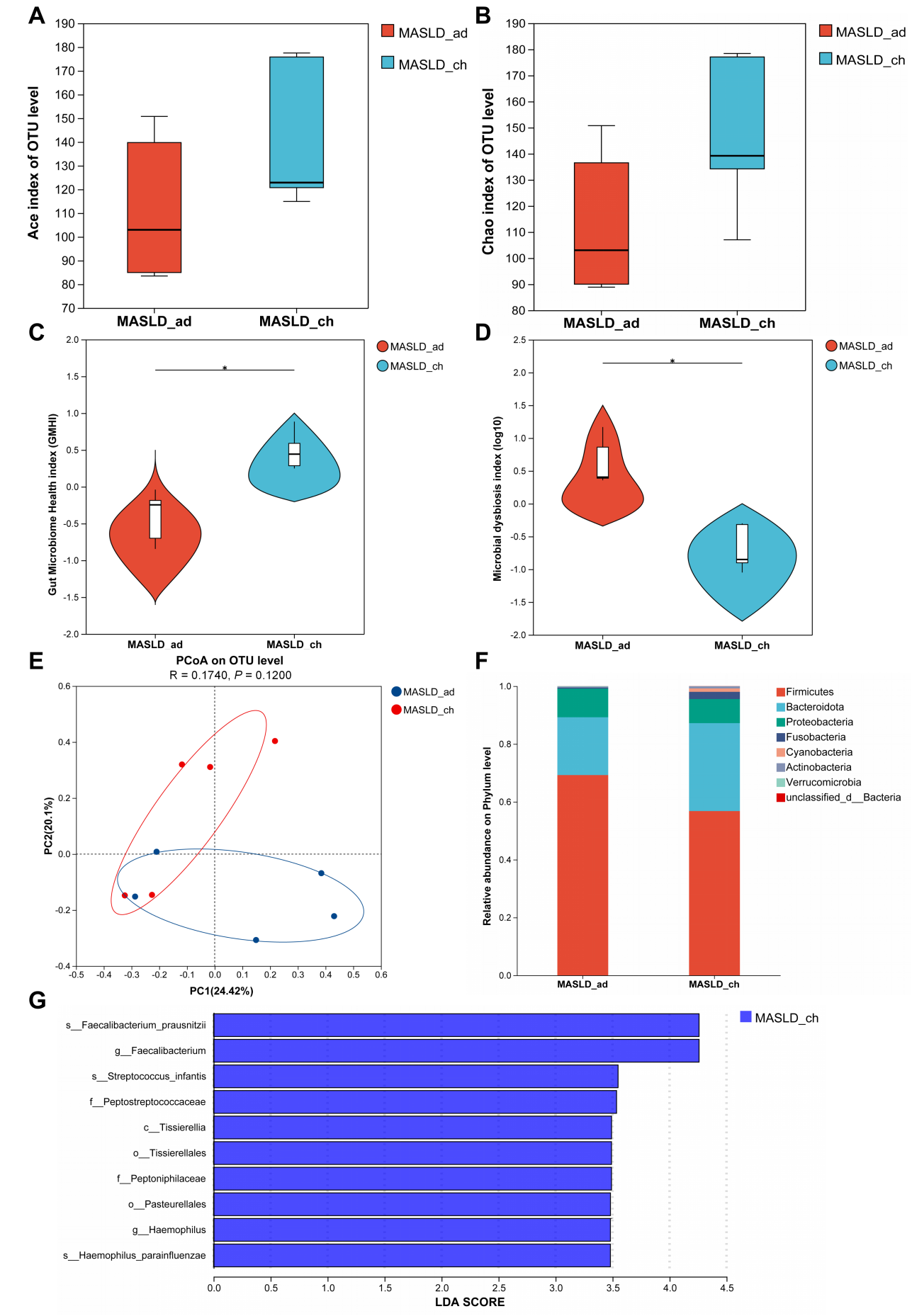


**Supplemental Figure 1. Alterations in the microbial diversity and gut microbiota composition associated with age in MASLD groups.** (A-B) The α-diversity measured by Ace index (A) and Chao index (B) in children (8-11 years, *n* = 5) and adolescents (12-16 years, *n* = 5) with MASLD. (C) Gut microbiome health index (GMHI) in the two groups. (D) Microbial dysbiosis index (MDI) was calculated for the two groups to assess the health states of the gut microbiota. (E) The principal coordinate analysis (PCoA) plot was used to show β-diversity among the groups at the OTU level. (F) Relative abundance of bacteria at the phylum level. (G) Linear discriminant analysis effect size (LEfSe) analysis of the gut microbiota in patients from the two groups. **P* < 0.05.

**Supplemental Figure 2**


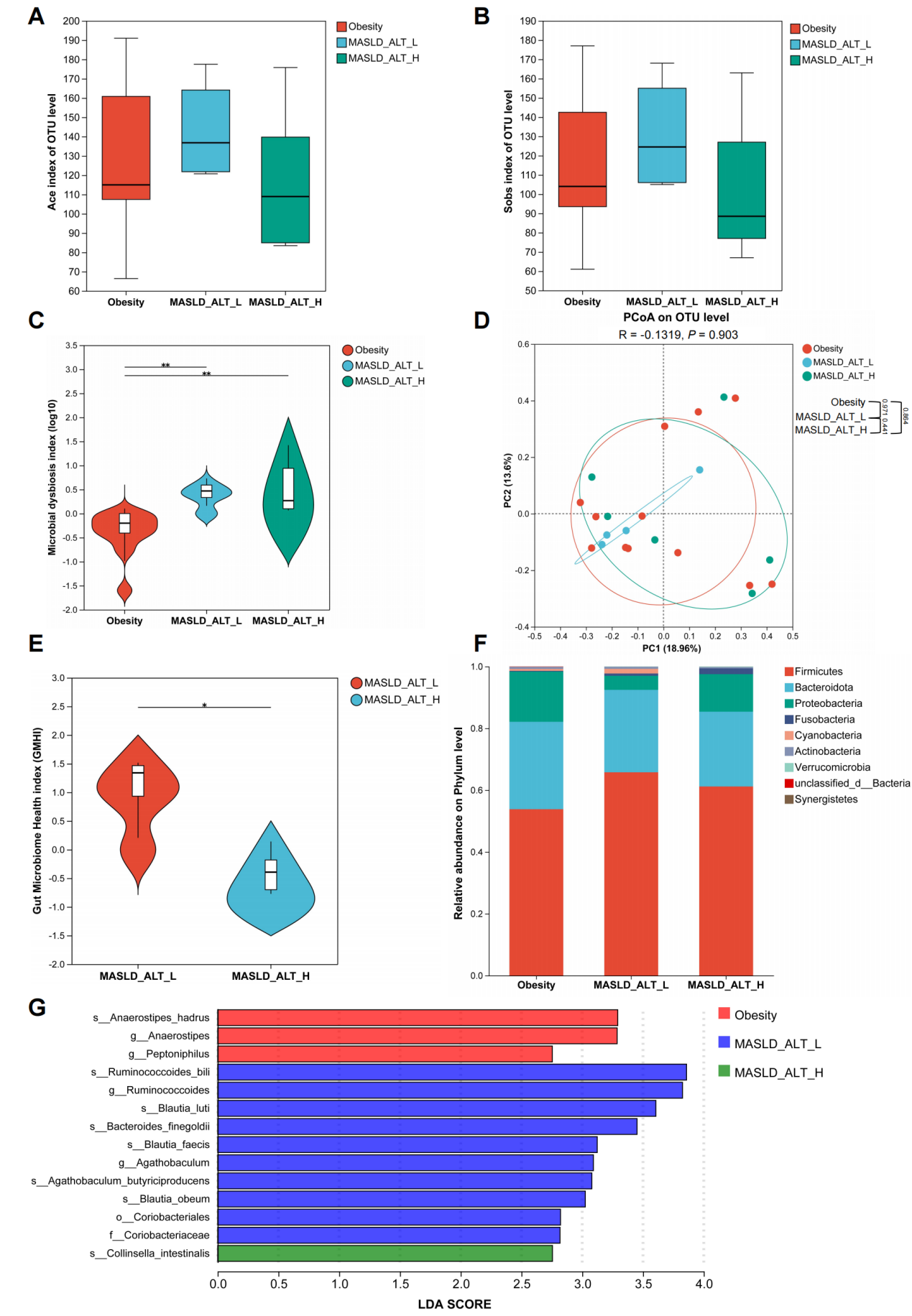


**Supplemental Figure 2. Alterations in the microbial diversity and gut microbiota composition associated with alanine aminotransferase (ALT).** (A-B) The α-diversity was evaluated by the Ace index (A) and Sobs index (B) in children with obesity (*n* = 12), MASLD with low ALT (*n* = 4; ALT < 50 U/L for boys and 44 U/L for girls), and MASLD with high ALT (*n* = 6; ALT ≥ 50 U/L for boys and 44 U/L for girls). (C) Microbial dysbiosis index (MDI) was calculated for the three groups. (D) The principal coordinate analysis (PCoA) plot was used to show β-diversity among the groups at the OTU level. The PERMANOVA *P* value was calculated with 999 permutations. (E) Gut microbiome health index (GMHI) in children with MASLD. (F) Gut microbiota composition at the phylum level in children with obesity, MASLD with low ALT, and MASLD with high ALT. (G) Linear discriminant effect size analysis (LEfSe) was performed to identify bacteria most likely to account for differences among the three groups from the phylum level to the species level. **P* < 0.05, ***P* < 0.01.
